# Acute Cardiac Failure due to Intra-Atrial Mass Caused by Zygomycetes in an Immunocompromised Paediatric Patient

**DOI:** 10.1155/2010/241791

**Published:** 2010-07-04

**Authors:** G. J. van de Glind, C. E. M. Gidding, C. M. W. Verlaat, K. Duthoi, A. P. C. Backx, P. E. Verweij, A. Warris

**Affiliations:** ^1^Department of Pediatrics, Radboud University Nijmegen Medical Centre, P.O. Box 9101, 6500 HB Nijmegen, The Netherlands; ^2^Department of Pediatric Intensive Care, Radboud University Nijmegen Medical Centre, P.O. Box 9101, 6500 HB Nijmegen, The Netherlands; ^3^Department of Pathology, Radboud University Nijmegen Medical Centre, P.O. Box 9101, 6500 HB Nijmegen, The Netherlands; ^4^Department of Medical Microbiology, Radboud University Nijmegen Medical Centre, P.O. Box 9101, 6500 HB Nijmegen, The Netherlands; ^5^Nijmegen Institute for Infection, Inflammation and Immunity, Radboud University Nijmegen Medical Centre, P.O. Box 9101, 6500 HB Nijmegen, The Netherlands

## Abstract

Cardiac zygomycosis can be a critical condition with sudden onset of severe congestive heart failure followed by severe hemodynamic deterioration. We report a fatal course of disseminated fungal infection with a massive intra-atrial thrombosis caused by a zygomycete, in a five year old boy treated for acute lymphoblastic leukaemia. In addition, we discuss the literature concerning infections caused by zygomycetes involving the heart. Prognosis is poor. A high index of suspicion and an aggressive diagnostic and therapeutic approach with the prompt start of preemptive antifungal therapy are key factors to improve outcome.

## 1. Introduction

Zygomycosis is an opportunistic infection caused by fungi of the class *Zygomycetes *[1, 2]. It is transmitted by inhalation, via a variety of percutaneous routes, or by ingestion of spores, with rhinocerebral and pulmonary diseases being the most common manifestation. Invasive zygomycosis is associated with angioinvasive disease, leading to thrombosis and infarction of involved tissues. The Zygomycota are generally characterised by formation of wide ribbon-like aseptate hyaline hyphae and their sexual reproduction with formation of zygospores [2].

In hematologic neutropenic patients zygomycosis is frequently characterised by disseminated disease and a rapidly fatal course. One of the organs that can be infected is the heart. A review of autopsy findings in 60 patients, with various underlying conditions (cardiac surgery, neoplasia, renal failure, intravenous drug use) and cardiac fungal infections reported the discovery of a zygomycete in 12% of cases [3]. Acute fulminant cardiac zygomycosis is a critical clinical condition with sudden onset of severe congestive heart failure followed by severe hemodynamic deterioration. 

We report a fatal course of disseminated fungal infection with cardiac involvement caused by a zygomycete in a five year old boy treated for acute lymphoblastic leukaemia (ALL). Paediatric ALL patients are considered to have a low risk for developing invasive mould infections. Persistent signs of infection in these patients without a causative agent should not take for granted but instead extensive and invasive diagnostic procedures should be performed to be able to target antimicrobial therapy.

## 2. Case Presentation

A five year old boy, diagnosed at the age of four with an ALL without central nervous system involvement or unfavourable cytogenetic features, presented with a history of fever, neutropenia and a painful right leg since four days and a right lower lobe consolidation with unilateral pleural effusion on chest X-ray. At that time he was treated for one year according to Dutch Childhood Oncology Group (DCOG) ALL-10 intermediate risk protocol. Due to persisting fever after 4 days of broad-spectrum antibiotics (ceftazidime and teicoplanin), the negative blood cultures and the abnormal findings on chest radiography, the patient was admitted to our hospital.

Physical examination at this time revealed no abnormal cardiac sounds, no abnormal breathing sounds, no hepatic or splenic enlargement, no lymphadenopathy and no signs of inflammation of the right leg. Laboratory studies showed a haemoglobin value of 6.3 mmol/l (after blood transfusion) and a leuco- and thrombocytopenia (leucocytes 0.6 × 10^9^/l; thrombocytes 50 × 10^9^/l). Blood chemistry showed an elevated C-reactive protein (CRP 145 mg/l). 

Additional diagnostic studies to detect a causative agent were done. Blood cultures taken were repeatedly negative for bacteria and fungi. Urine culture and faeces were negative for fungal pathogens. The multiple sputum cultures taken did not reveal any microorganisms. Cultures of both pleural fluid obtained by transthoracic puncture and broncho-alveolar lavage (BAL) were negative for viral, bacterial and fungal agents. *Aspergillus*-antigen (galactomannan) was not detected in multiple plasma samples, pleural fluid and BAL fluid. Bone marrow aspirate to rule out a relapse of his leukaemia showed no malignant cells and cultures for bacteria and mycobacteria were negative. Urine tested negative for *Legionella *antigen. Ciprofloxacin was added to the antimicrobial therapy, to cover atypical causative agents of pneumonia. Empiric anti-fungal therapy was not initiated because of the stable condition of the patient and the negative test results at that moment.

Despite broad-spectrum antibiotic treatment (ceftazidime, teicoplanin and addition of ciprofloxacin for two days), fever persisted although the clinical condition remained stable. After 7 days, the developed a hepatomegaly and blood tests results showed still a pancytopenia, an elevated CRP (192 mg/l) and increased liver enzymes. Ultrasound examination of the abdomen confirmed the hepatomegaly without any focal lesions which might have been suggestive for fungal infection. 

**Figure 1:**
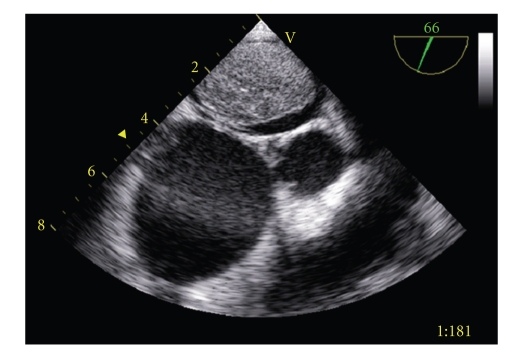
Ultrasound of the heart showing a round left intra-atrial tumour mass, compromising almost the whole left atrium.

 On day 10 he was transferred to the paediatric intensive care unit (PICU) of our hospital because of progressive respiratory distress and signs of a capillary leakage syndrome. At this time, the urine and faeces cultures became positive for *Candida glabrata*, while one out of many sputum samples grew *Aspergillus fumigatus*. Physical examination at this time showed a pale, moaning boy in respiratory distress needing supplementary oxygen via a non-rebreathing mask. On the nail of digit III of the left foot and on the soles of both feet small vasculitis-like lesions were noticed. Because of imminent respiratory failure the patient was intubated and artificial ventilated. Chest X-ray showed the known consolidation in the right lower lobe, but also pulmonary oedema with haziness of the perihilar regions. Transoesophageal ultrasound examination of the heart showed hypocontractility, with a round left intra-atrial tumour mass, compromising almost the whole left atrium (Figure 1). Suspicion of a fungal mass or infected thrombus together with the recovery of fungal pathogens in urine, faeces and sputum, led to the initiation of antifungal therapy with liposomal amphotericin B. Progressive hemodynamic failure developed without an effect of high-dose inotropic medication and the clinical condition of the patient deteriorated fast. Several hours after admission to the PICU the patient died.

## 3. Autopsy Findings

**Figure 2:**
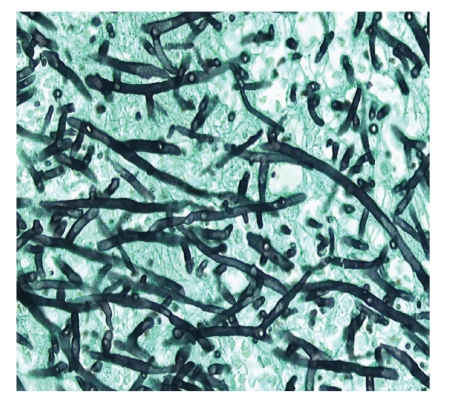
Histology of the intracardial mass showing a great amount of fungal hyphae, characterised by irregular wide, non-septated hyphae with right angle branching, consistent with zygomycetes (Grocot stain, magnification 40x).

Autopsy revealed zygomycosis with septic embolisms in almost all organs. The most striking lesion was a thrombus in the left atrium of the heart. It was firm, grey-white of colour and had a diameter of 5 cm. It was confirmed to the right pulmonary vein and reached towards the mitralic valve. The microscopic findings showed a great amount of fungal hyphae embedded in a fibrin-rich medium (Figure 2). In addition some coronary arteries were filled with septic emboli and had led to multifocal necrosis of the heart muscle. The right lung was doubled in weight with a severe damaged right middle lobe. Microscopically, the lobe was totally ischemic, with haemorrhage and oedema. It was caused by several septic embolisms in the medium-sized and minor vessels where the fungus invaded the arterial wall and penetrated into the interstitial tissue. 

The diagnosis of cardiac zygomycosis with septic embolisms was based on the morphology of the hyphae found in almost all the organs. The hyphae were typically irregularly wide, non-septated and showed frequent right angle branching. At autopsy tissue samples from liver, spleen and blood were taken but zygomycetes were not recovered. Unfortunately, no cultures were obtained from the right lung, heart and intracardial mass. Post-mortem blood cultures were positive for *C. glabrata*.

## 4. Discussion

In patients with haematological malignancies, zygomycosis is frequently characterised by disseminated disease and a rapidly fatal course. Although a reduction in mortality has been observed recently, the mortality rate still remains high (70%) [4]. Outcome varies as a function of the underlying condition (mortality rate up to 66% in malignancy), site of infection (mortality rate up to 96% in disseminated disease) and use of antifungal therapy (survival in 70% of patients treated with antifungal therapy and surgery in contrast to 3% in patients that were not treated) [5]. A recent study reports an overall mortality of zygomycosis in solid organ transplant recipients of 49%, with disseminated and rhinocerebral zygomycosis having the poorest prognosis. Surgery combined with amphotericin B (especially the liposomal formulation) showed to be associated with a better outcome [6]. The high mortality rate can be explained partly by diagnostic difficulties. In neutropenic patients ante mortem diagnosis is uncommon because blood cultures are seldom positive and the diagnostic yield of sputum cultures and bronchial washings is limited. Furthermore, there are currently no biological markers available that detect zygomycetes. Nosari et al. obtained a diagnosis in adults in vivo only by invasive procedures, like biopsy or surgery [7]. 

 We conducted a literature search and identified forty-six cases of zygomycosis with cardiac involvement reported in English-written literature until 2009 [6, 8–41], including five children aged 2, 8, 9, 13 and 14 years [10, 20, 36, 40]. Underlying disorders were hematological/oncological in 19 patients, including 3 children. Development of intra-cardiac thrombi occurred in 14 cases. The overall mortality in this patient group was 89% (41 of 46 cases). In the five patients who survived zygomycosis with cardiac involvement, zygomycosis was restricted to the heart, all went through invasive diagnostic procedures and intensive treatment including the combination of surgery and antifungal therapy [25, 29, 34, 36, 40]. The pathogenesis is not clear in our patient. Autopsy findings showed infected pulmonary vasculature, and no evidence for pneumonia. The infected intra-cardiac tumour mass may have originated from a cardiac valve. Children with congenital heart defects, with surgery using vascular patches and grafts, or patients with valvular disease, with intravenous devices and antibiotic use, are at greater risk for a fungal endocarditis [42, 43]. Our patient had a vascular line in situ with its tip lying in the right atrium. Perhaps this could have been the porte d'entrée, as recent studies showed a lack of concordance between site of endothelial damage and localisation of fungal endocarditis [44]. 

More extensive diagnostic procedures could have resulted in earlier identification of the causative agents. Nosari et al. found prompt chest CT scan helpful (in 7/13 adult patients) in diagnosing filamentous fungal infections [7]. In our patient, a CT-thorax was not performed, because the chest X-ray showed very clearly the presence of a pulmonary infiltrate. Retrospectively, a lung biopsy should have been performed after obtaining the negative results of the pleural and BAL fluid examinations. Lass-Flörl and her colleagues showed that CT-guided lung biopsy specimens provided reliable tools for diagnosing invasive fungal infections [45]. Especially the performance of the calcofluor white staining to differentiate between septate and unseptate hyphae helped guide decisions regarding adequate therapy. 

 It is questionable if there was a reason to perform at an earlier stage a cardiac ultrasound. An intra-cardiac tumour mass caused by fungal infection is rare and has a low rate of suspicion. In addition, no abnormal cardiac sounds were heard on physical examination. 

With the availability of a wider arsenal of antifungal agents and the changing epidemiology of fungal pathogens that cause invasive fungal disease, the importance of identification of the infecting fungal pathogen has increased. Voriconazole, being first-line treatment for invasive aspergillosis, the most common encountered invasive filamentous fungal infection in our hospital, has no activity against *Zygomycetes* [46–48], neither has caspofungin [49]. Lipid formulations of amphotericin B remain the gold standard in the treatment of mucormycosis [2, 7]. Posaconazole, a new triazole antifungal, has potential benefits for patients with mucormycosis. Is has shown in vitro activity against zygomycetes [49–51] as well as in vivo effectiveness [52]. By reviewing the literature, only those patients undergoing intensive treatment, with a combination of surgery and amphotericin B (with or without other antifungal agents), survived. 

 Retrospectively, this case report illustrates two important aspects of diagnosing and treating invasive fungal infections in immunocompromised patients. First, extensive efforts should be made to localise the infection and its extensiveness, followed by invasive procedures to detect the causative fungus. Second, if moulds are suspected and no signs of invasive aspergillosis (i.e. positive galactomannan test, septate hyphae in tissue) are present, one should reconsider if voriconazole should be the first choice treatment. As demonstrated in this case report, lipid formulations of amphotericin B should be the first choice to cover a broader range of fungal pathogens.
